# An Agreeable Antiseptic Tooth-Powder

**Published:** 1886-10

**Authors:** 


AN AGREEABLE ANTISEPTIC TOOTH-POWDER.—

| | | | |
|---|---|---|---|
| ℞ | Boric acid, pulv., - - - | grs. xl. | |
| | Potass. chlor., - - - - | ʒ ss. | |
| | Pulv. Guaiaci, - - - - | grs xx. | |
| | Creæ Prep., - - - - | ʒ i. | |
| | Magnesiæ Carb., pulv., - - - | q. s. ad ℥i | |
| | Otto of Roses, - - - - | gtt. ss. | M. |

